# Multicenter study to describe viral etiologies, clinical profiles, and outcomes of hospitalized children with severe acute respiratory infections, Egypt 2022

**DOI:** 10.1038/s41598-023-48814-x

**Published:** 2023-12-09

**Authors:** Amr Kandeel, Manal Fahim, Ola Deghedy, Wael H. Roshdy, Mohamed K. Khalifa, Rabeh El Shesheny, Ahmed Kandeil, Saly Wagdy, Amel Naguib, Salma Afifi, Khaled Abdelghaffar

**Affiliations:** 1 Preventive Sector, Ministry of Health and Population, Cairo, Egypt; 2 Central Public Health Laboratories, Ministry of Health and Population, Cairo, Egypt; 3 Centre of Scientific Excellence for Influenza Viruses, National Research Centre, Dokki, Giza, 12622 Egypt; 4 Consultant Ministry of Health and Population, Cairo, Egypt; 5 Ministry of Health and Population, Cairo, Egypt

**Keywords:** Epidemiology, Risk factors

## Abstract

In late 2022, severe acute respiratory infections (SARI) surveillance reported an abrupt increase in non-COVID-19 infections among children after three years of drastic reductions. Signals of increased absenteeism due to respiratory symptoms among primary and preparatory school children were detected by Event-Based Surveillance. We conducted a hospital-based survey of children who were admitted with SARI to identify the causative pathogen(s) and estimate the burden of infection. A survey was conducted among children < 16 years in 21 referral hospitals in the three governorates with the highest SARI rates. Patients’ demographics, clinical symptoms, and severity were collected from medical records using a line list. Patients were swabbed and tested for a panel of 33 respiratory pathogens by RT-PCR at the Central Laboratory in Cairo. Descriptive data analysis was performed for demographic data. Patients’ characteristics were compared by causative agents’ clinical picture and severity using Chi2 with a *p* < 0.05 significance. Overall, 317 patients were enrolled, 58.3% were ≤ 1 year of age, 61.5% were males. Of 229 (72.7%) of positively tested patients, viruses caused 92.1% including RSV 63.8%, Rhinovirus 10.0%, Influenza 9.2%, Adenovirus 5.2%, and 1.3% co-infected with two viruses. Bacteria caused 3.5% of cases and 4.4% had mixed viral-bacterial infections. Rhinovirus was the most common cause of death among children with SARI, followed by RSV (8.7% and 1.4%), whereas influenza and Adenovirus did not result in any deaths. Patients with viral-bacterial infections are more likely to be admitted to ICU and die at the hospital than bacterial or viral infections (60% and 20% vs. 31.8% and 1.9% vs. 12.5% and 12.5%, *p* < 0.001). Viruses particularly RSV are the leading cause of SARI causing significant health problem among children < 16 years in Egypt. Bacterial on top of viral infection can worsen disease courses and outcomes. Studies are required to estimate the SARI burden accurately among Egyptian children and a comprehensive approach tailored to Egypt is necessary to reduce its burden.

## Introduction

Globally, during the COVID-19 pandemic, there was a significant reduction in the transmission of several common viral respiratory pathogens, resulting in fewer hospitalizations and deaths in children and adults caused by these viruses. The viruses with reduced transmission during the COVID-19 pandemic include endemic Human Coronaviruses, human metapneumovirus (HMPV), Parainfluenza viruses (PIV), Respiratory Syncytial virus (RSV), and influenza, whereas adenovirus and rhinovirus circulation was less affected^1–3^. Research suggests that nonpharmaceutical preventive measures, competition within the host, and population-level interactions reduced the circulation of common respiratory viruses during the COVID-19 pandemic^3,4^.

Acute respiratory infections (ARIs) are a leading cause of morbidity and mortality among children under five, particularly in developing countries, and pose a major threat to healthcare systems^8^. In Egypt, ARIs are monitored through integrated surveillance for ARI, National Disease Surveillance System for communicable diseases (NEDSS), ARI syndromic surveillance, ARI mortality surveillance, and event-based surveillance systems (EBS)^9^. ARI's integrated surveillance system monitors the activity of influenza and SARS-CoV-2 at 12 influenza like illness (ILI) and 17 severe acute respiratory infections (SARI) sentinel sites located in 19 governmental hospitals, plus the Central Public Health Laboratory (CPHL) and a network of five regional laboratories. Surveillance started to monitor RSV in 2016 among children less than two years to better understand the epidemiology and disease trends in Egypt^10^. The surveillance reported a drastic reduction in influenza and RSV activity early in the COVID-19 pandemic, which persisted in varying degrees throughout, a finding noted by other researchers^7,10^. However, surveillance detected a surge of both viruses by the end of 2022 reported by many governorates. In addition, there were signals of increased school absenteeism due to respiratory symptoms among primary and preparatory school children caught by EBS in different Egyptian governorates^10^.

A recent increase in non-SARS-CoV-2 infections have been reported across different climate zones and regions, suggesting transmission levels may return to pre-pandemic levels after COVID-19 mitigation practices are relaxed^5,6^. It is unclear how the ongoing pandemic will affect the circulation of common respiratory viruses given the continuous changes in the activity and immunity to SARS-CoV-2 and these viruses^7^. This study aims at evaluating the impact of the pandemic on the transmission of seasonal non-SARS-CoV-2 respiratory viruses in Egypt.

## Methods

MoHP conducted a national survey to identify the pathogen(s) responsible for the increased transmission of SARI among Egyptian children under the age of 16, as well as to describe the clinical picture, severity, and outcome of SARI-causative agents.

### Study setting and population

A hospital-based survey was conducted between 1 and 15 November 2022 at 21 hospitals, seven in each governorate, including infectious diseases, chest, and general hospitals in Cairo, Giza, and Gharbia, the three governorates with the most reported cases of SARI (unpublished data from the national surveillance system). Cairo is Egypt's capital and largest urban governorate with about 10 million residents. Giza, a semi-urban governorate with many slum areas, is the second largest governorate situated near Cairo with about 7 million people and Gharbia is a mostly rural governorate 90 km north of Cairo with 5 million residents.

Study participants were children < 16 years of age currently admitted to inpatient wards, intermediate care units, and neonatal intensive care units of the selected hospitals with SARI symptoms including a history of/ or measured fever of ≥ 38 °C and cough.

### Sample size calculation

According to the surveillance, the prevalence of influenza among hospitalized patients with SARI is estimated to be 12%. Based on the National Census of 2022, Egypt has about 35 million children < 16 years of age^11^. With a precision of 20%, a confidence limit of 95%, and a design effect of 2, at least 325 patients were required to provide a representative sample.

### Data collection

A line list containing patients’ demographics, clinical symptoms, disease onset date, admission date, preliminary admission diagnosis, the presence of comorbidities, ICU admission, and patient status on discharge was developed. The data was collected from patients’ medical records, and patient/guardian interviews if needed.

### Laboratory procedures

Oropharyngeal and Nasopharyngeal (NP/OP) swabs were collected from all study participants using a dry sterile tip flocked with a nylon fiber swab applicator to swab the posterior pharynx and the nostril for OP and NP swabbing respectively. Specimens were stored in viral transport media (VTM) and shipped to the Central Public Health Laboratory (CPHL) in nitrogen tanks or ice box according to the distance, each specimen was tested for 21 respiratory viruses by Real-time polymerase Chain Reaction (RT-PCR) (FTD Respiratory Pathogens 21 Assay, Seimens, Germany). The respiratory panel 21 can detect the following viruses (influenza A, influenza A H1N1 influenza B, human respiratory syncytial virus A and B, parainfluenza virus 1, 2, 3, 4, coronavirus 229E, HKU1, NL63, OC43, Picornaviruses, rhinovirus, enterovirus, parecho virus, Human metapneumovirus A and B.

For detection of bacterial pathogens 2–3 ml ETDA blood samples were collected and shipped to CPHL using ice boxes, samples were extracted using The chemagic™ 360 instrument for automated DNA purification . Multiplex RT-PCR technique for bacterial panels (Fast Track Diagnostics RT- PCR Respiratory pathogens 33 Assay) (Siemens, Germany) was used to detect invasive respiratory pathogens. The panel included *Bordetella *spp.,* Chlamydophila pneumoniae, Hemophilus influenzae, Klebsiella pneumoniae, Legionella pneumophila/longbeachae, Moraxella catarrhalis, Mycoplasma pneumoniae, Pneumocystis jirovecii, Salmonella *spp., *Staphylococcus aureus and Streptococcus pneumoniae*^12,13^.

### Statistical analysis

Data analysis was conducted using SPSS software (version 28.0). Data were summarized using mean and standard deviation for quantitative variables whereas numbers and percentages were used for qualitative variables. Comparison between groups was done using the Chi-square test for qualitative variables, the t-test for normally distributed quantitative variables, and the Mann–Whitney test for abnormally distributed data.

### Ethics approval statement

The study was approved by the Ministry of Health and Population Institutional Review Board" (IRB) as a minimal-risk research study. All procedures were carried out according to the Ministry of Health and Population Institutional Review Board guidelines and regulations.

### Patient consent statement

There was a minimal risk associated with this study, which involved only the collection of medical history and symptoms, while specimens and laboratory tests were performed as part of routine patient management, not for study purposes. In order to participate in the study, written informed consent was obtained from the parents/legal guardians of the patient for participation in the study.

## Results

Overall, 317 patients were enrolled from the 21 hospitals with a mean number of patients per participating hospital being 14 (range 4–33) patients. Patients’ median age was 8.1 months [IQR = 3.8–24.3 months], with more than half of them (58.3%) ≤ 1 year of age, and 61.5% were males. Approx. half of the patients 137 (43.2%) were residents of Giza governorate, 93 (29.3%) of Cairo, and 87 (27.5%) were from Gharbia. Of all patients, 37 (11.7%) have comorbidities including chronic chest disease (43.2%), cardiovascular (21.6%), neurologic (16.2%), immunocompromised (13.5%), and 5.4% other chronic diseases. Three fourth of them (75.1%) had pneumonia and 10 (3.2%) patients died at the hospital.

A total of 229 (72.2%) of the patients tested positive for one or more respiratory pathogens. Of these, 208 (90.8%) had a single viral infection, 3 (1.3%) had two viral coinfection, 8 (3.5%) had bacterial infection, and 10 (4.4%) had mixed bacterial and viral infection. The most prevalent viral cause was RSV with 146 patients representing 63.8% of all positively tested patients, followed by rhinovirus 23 (10.0%), influenza 21 (9.2%), and adenovirus 12. Furthermore, three patients were infected by OC43, two parainfluenza viruses, and one human metapneumovirus, and three patients were coinfected with two viruses. In the 21 patients with influenza viral infection, 15 (71.4%) had subtype A/H3N2, 4 (19.0%) had untypable influenza A, one had A/H1N1, and one had influenza B. Of the eight patients with bacterial causes six had Hemophilus influenza (non-type B) and two had Streptococcus pneumoniae. Of the ten patients who had mixed viral-bacterial pathogens, seven had RSV, two Rhinovirus, and one Adenovirus, with half of them coinfected with Streptococcus pneumoniae (Table 1).

**Table 1:** Viral and bacterial causes of severe acute respiratory infections among Egyptian children < 16 years of age admitted to 21 hospitals in three Egyptian governorates.

Causative agents	Number	Percent
Total patients tested	**317**	
Single virus infections	**208**	**90.8**
Respiratory Syncytial Virus (RSV)	146	63.8
Rhinovirus	23	10.0
Influenza	21	9.2
Adenovirus	12	5.2
Coronavirus (OC43)	3	1.3
Parainfluenza	2	0.9
Human Metapneumovirus	1	0.4
Viral-viral coinfections	**3**	**1.3**
Rhinovirus- Adenovirus	1	0.4
Rhinovirus- Influenza-A	1	0.4
Rhinovirus- Influenza-B	1	0.4
Bacterial infections	**8**	**3.5**
*H. influenzae* (non-type B)	6	2.6
*S. Pneumoniae*	2	0.9
Viral-bacterial mixed infection	10	4.4
RSV- H. influenzae (non-type B)	4	1.7
RSV- S. Pneumoniae	2	0.9
Adenovirus- *S. pneumoniae*	1	0.4
Rhinovirus- S*. pneumoniae*	1	0.4
SARS-CoV-2- Rhinovirus- *H. influenzae* (non-type B)	1	0.4
RSV- S. Pneumoniae–Moraxella	1	0.4

*RSV * Respiratory Syncytial virus, *H. influenza* *Hemophilus influenzae* (non -type B), *S. Pneumoniae* *Streptococcus pneumoniae*.

Significant values are in [bold].

It was found that RSV and Adenovirus infect mainly infants and young children (71.0% and 58.3% were under 1 year), while influenza and Rhinovirus tend to infect older children (71.4% and 73.9% > 1 year). while no differences were observed between gender and positive viral results (*p* = 0.6). According to governorates of residence, Adenovirus and RSV were most prevalent in Giza (83.3% and 46.6% of patients respectively) whereas Rhinovirus and influenza were most prevalent in Gharbia (69.6% and 57.1% of patients respectively) (Table 2).

**Table 2:** Comparison between the main types of viral infections in hospitalized children < 16 years of age, Egypt National acute respiratory infections survey, 2022.

Characteristics	RSV (n = 146)		Rhinovirus (n = 23)		Influenza (n = 21)		Adenovirus (n = 12)		*p* value
	No.	%	No.	%	No.	%	No.	%	
Mean age ± SD (years)	1.1 ± 1.9		3.0 ± 3.6		3.3 ± 3.1		2.6 ± 3.9		< 0.001*
Age groups									
< 1 month	8	5.5	2	8.7	0	0.0	1	8.3	< 0.001*
1month-1year	95	65.5	4	17.4	6	28.6	6	50.0	
> 1–5 years	36	24.8	13	56.5	10	47.6	3	25.0	
> 5 years	6	4.1	4	17.4	5	23.8	2	16.7	
Gender									
Male	85	58.2	15	65.2	12	57.1	9	75.0	0.6
Female	61	41.8	8	34.8	9	42.9	3	25.0	
Governorate of residence									
Giza	68	46.6	0	0.0	5	23.8	10	83.3	< 0.001*
Cairo	47	32.2	7	30.4	4	19.0	2	16.7	
Gharbia	31	21.2	16	69.6	12	57.1	0	0.0	
Symptoms									
Fever	22	15.1	9	39.1	10	47.6	1	8.3	0.23
Cough	124	84.9	20	87.0	21	100.0	11	91.7	0.26
Sore throat	22	15.1	9	39.1	10	47.6	1	8.3	< 0.001*
Dyspnea	129	88.4	17	73.9	9	42.9	9	75.0	< 0.001*
Vomiting	19	13.0	0	0.0	1	4.8	5	41.7	< 0.01*
Diarrhea	22	15.1	1	4.3	1	4.8	3	25.0	0.19
Rhinorrhea	37	25.3	0	0.0	1	4.8	3	25.0	< 0.01*
Comorbidity	9	6.2	5	21.7	2	9.5	3	25.0	0.02*
Severity indicators									
Pneumonia	110	75.3	15	65.2	13	61.9	9	75.0	0.46
ICU admission	52	35.6	8	34.8	4	19.0	1	8.3	0.13
Died at hospital	2	1.4	2	8.7	0	0.0	0	0.0	0.19

RSV and Adenovirus showed a similar clinical picture with higher rates of vomiting, diarrhea, and rhinorrhea (13.0%, 15.1%, 25.3%, 41.7%, 25.0%, and 25.0%). In contrast, influenza and Rhinovirus were associated with higher rates of fever and sore throat (39.1%, 3,9.1,% and 47.6%, 47.6% respectively) (Table 2).

The highest rate of dyspnea was found in RSV patients (88.4%), followed by adenovirus (75.0%) and Rhinovirus (73.9%), whereas it was the lowest in influenza patients (42.9%). Adenovirus and Rhinovirus were more likely to infect children with comorbidities than RSV and influenza, which tend to infect normal healthy children (25.0% and 21.7% of patients vs. 6.2% and 9.5% of patients respectively *p* < 0.05). Rhinovirus was the most common cause of death among children with SARI, followed by RSV (8.7% vs. 1.4%), whereas influenza and Adenovirus did not result in any deaths (Table 2).

The study indicated that the majority of viral-bacterial infections affect infants under one year of age, while viral infections mostly affect children under five, while bacterial infections affect older children (mean ages = 0.5 ± 0.4, 1.7 ± 2.6, and 2.8 ± 4.3 respectively). Patients with viral-bacterial infections had insignificantly higher rates of severe symptoms including fever, dyspnea, and pneumonia (80.0, 100.0, and 80.0%) compared to the viral and bacterial infections, in addition, they are more likely to be admitted to ICU and die at the hospital than bacterial and viral causes (60.0 vs 12.0 vs 31.0% and 20.0 vs 12.5 vs 1.9% respectively). In children with comorbid conditions, a bacterial infection is more common than viral or viral-bacterial infection (37.5% vs. 9.5%, and 10%, *p* < 0.04) (Table 3).

**Table 3:** Comparison between viral, bacterial and viral-bacterial mixed infections among hospitalized children < 16 years of age, Egypt National acute respiratory infections survey, 2022.

	Viral (n = 211)		Bacterial (n = 8)		Mixed Viral-bacterial (n = 10)		*p* value
Mean age in years	1.7 ± 2.6		2.8 ± 4.3		0.5 ± 0.4		0.20
Age group							
< 1 month	13	6.2%	1	12.5%	2	20.0%	0.37
1 month-1year	112	53.1%	3	37.5%	7	70.0%	
> 1–5 years	65	30.8%	3	37.5%	1	10.0%	
> 5 years	21	10.0%	1	12.5%	0	0.0%	
Gender							
Male	129	61.1%	5	62.5%	6	60.0%	0.99
Female	82	38.9%	3	37.5%	4	40.0%	
Governorate of residence							
Giza	83	39.3%	5	62.5%	6	60.0%	0.09
Cairo	62	29.4%	3	37.5%	4	40.0%	
Gharbia	66	31.3%	0	0.0%	0	0.0%	
Symptoms							
Fever	159	75.4%	5	62.5%	8	80.0%	0.66
Cough	185	87.7%	6	75.0%	9	90.0%	0.55
Sore throat	44	20.9%	1	12.5%	0	0.0%	0.23
Dyspnea	171	81.0%	5	62.5%	10	100.0%	0.13
Vomiting	25	11.8%	1	12.5%	0	0.0%	0.51
Diarrhea	27	12.8%	0	0.0%	0	0.0%	0.27
Rhinorrhea	41	19.4%	0	0.0%	1	10.0%	0.30
Comorbidity	20	9.5%	3	37.5%	1	10.0%	0.04
Severity							
Pneumonia	155	73.5	4	50.0	8	80.0	0.30
ICU admission	67	31.8	1	12.5	6	60.0	< 0.001*
Died at hospital	4	1.9%	1	12.5%	2	20.0%	0.001*

## Discussion

During the past three years, SARS-CoV-2 dominated most respiratory viruses because of strict respiratory precautions; it is expected that common respiratory viruses will return to their pre-pandemic levels as COVID-19 mitigation practices loosen^7^. Worldwide, ARIs are one of the leading causes of childhood morbidity and mortality^14^. This survey study provides insights into the causes of severe acute respiratory infections among hospitalized children age in Egypt while the world is transitioning out of the COVID-19 pandemic. Additionally, it compares the clinical picture and outcomes of infections caused by viruses, bacteria, and viral-bacterial mixed infections.

According to the study, viruses account for the majority of cases of SARI in children under 16 years old, while bacteria and mixed viral-bacterial causes account for a small percentage. Similarly, several studies have found that viruses are more likely to cause the majority of upper and lower respiratory tract infections in children^15–18^. However, these studies reported higher rates of bacterial and mixed viral-bacterial infections among children with SARI compared to this study.

Several European countries reported early and intensified RSV transmission in late 2022^5^. The United States and Canada reported significant increases in RSV cases, warning of a triple demic that will mostly affect children^16^. The RSV surge in the northern hemisphere began unseasonably early causing severe disease courses so pediatric hospitals experienced problems because of the number of children requiring hospital admission^20–22^. RSV respiratory infection was associated with substantial morbidity even in high-income counties, resulting in the hospitalization of one in 56 healthy term-born infants^23^. In accordance with this, this study found high rates of RSV infection among hospitalized children, especially among children under two who missed their first exposure to the virus due to the COVID-19 lockdown in the early years of the pandemic^24^.

In this study, Rhinovirus was found to be the second cause of SARI among Egyptian children, however, it was much less common than RSV. Studies in other countries found that Rhinovirus and Enterovirus were the most frequently detected viruses during the pre-and COVID-19 pandemic periods in hospitalized children^25^. Transmission of non-enveloped viruses such as Rhinovirus and Adenovirus was not affected by the COVID-19 pandemic in comparison to enveloped viruses such as RSV and influenza^26^. The difference was explained by virological, environmental, and behavioral factors that contributed to the stability of the non-enveloped viruses on surfaces and their transmission routes^27,28^. More research is needed to understand the factors contributing to the low transmission of enveloped viruses during the COVID-19 pandemic and the resurgence of these viruses following the pandemic recess.

Studies from Egypt indicated that viruses were the main causes of SARI in children < 16 years in the pre-pandemic phase with RSV and Adenovirus the being most common viruses and Influenza less common^29,30^. The same pattern was found in this study where influenza came third cause of SARI during the late phase of the pandemic. Influenza started to re-emerge as a cause of ARI in Egypt after two years of an unprecedented reduction in influenza transmission in 2019–2020. The re-emergence of influenza in Egypt began in 2020–2021 and continued throughout the 2022–2023 seasons, but the circulation level remained lower than the pre-pandemic levels^10^. During the recent resurgence of influenza, the H3N2 subtype was predominant, as shown in this study as well. Globally, influenza activity began to decline early in 2023, however, activity in the northern hemisphere remained high, with a higher proportion of H1N1pdm09 and H3N2 viruses circulating^6^. Continuous monitoring of the spread and severity of respiratory viruses at national and global levels is crucial to guide control measures and mitigate their impact^31^.

Research estimates that 99% of deaths in children under 5 years of age with influenza-related lower respiratory tract infections are found in developing countries^32^. In Egypt, severe disease with high fatality in younger healthy individuals was reported during 2020–2022, a finding also reported by Groves et al. in the 2020/2021 season^10,33^. However, no fatalities among children < 16 years of age with influenza was reported in this study. The current low CFR pattern in children with influenza reported in this study is close to what was observed in Egypt and other countries during the pre-pandemic period^34,35^. This could mark the return to influenza’s normal pattern of higher severity and fatality among older ages and in people with comorbidity^36^.

According to this study, RSV and adenovirus patients' demographic characteristics and clinical picture were similar, while influenza and rhinovirus patients' characteristics were more similar. Influenza and Rhinovirus affect older children who live in rural areas and could present with gastrointestinal symptoms in agreement with what was reported by Barati et al.^37^. In 2008. While RSV and Adenovirus infect younger children, who are residing in urban and suburban areas and usually present with respiratory symptoms including Sore throat and dyspnea, however, Adenovirus tended to occur in children with comorbidities while RSV tends to infect normal children, a finding also noted by Rajkumar et al.^38^. As a guide to diagnosing and managing SARI cases effectively, MoHP developed a clinical diagnosis and case management manual^39^. In addition, clinicians need to use the surveillance results to identify the circulating respiratory pathogens to help them decide on the appropriate treatment of ARIs.

We found that RSV and Rhinovirus infections were more severe than influenza and Adenovirus in terms of rate of pneumonia, ICU admission, and case fatality. Amini et al. reported a higher severity and fatality rate of RSV than influenza in children hospitalized with SARI^40^. Several studies also reported higher severity of Rhinovirus symptoms and clinical course especially among children with comorbid conditions, highlighting the role of rhinoviruses in severe ARI^25,41,42^. Early and accurate clinical and laboratory diagnosis of the viral cause of SARI in children is crucial for effective case management and positive disease outcome.

No patients of SARS-CoV-2 were identified among the Egyptian children in this survey, only one patient had mixed SARS-CoV-2, HI, and Rhinovirus infection. It was noted that children have constituted the lowest proportion of notified COVID-19 cases and Children often experience mild or asymptomatic COVID-19^43,44^.

Coinfection with influenza and SARS-CoV-2 was reported in Egypt before and led to more severe course and outcomes compared to a single viral infection^9,34^. Similarly, this study reported coinfection with more than one viral cause. Studies reported that approx. 10–30% of all respiratory viral infections are caused by more than one virus and it is common in children^45^. The low number of viral respiratory coinfections with SARS-CoV-2 among Egyptian children found in this survey could be attributed to the near absence of SARS-CoV-2. Some studies have found that respiratory viral coinfections have a severe outcome in children^46^, others have found no association, and some have found it does not contribute to the burden of the disease and may even protect against severe disease^34,47^. Further studies are needed to better identify the impact of respiratory viral coinfections on children.

A recent study indicated that viral-bacterial infection is more common in young children, this study reported the same finding^48^. Researchers suggested that it is caused by secondary bacterial infection on top of a primary viral infection^49^, while others suggested that prior colonization with pathogenic bacteria may enhance respiratory viral infections^48^. RSV-pneumococcal infection has been reported before, RSV has been shown to increase pneumococcal pneumonia in infants by 20–31%^50,51^. In addition, researchers reported a significant decline in the number of hospitalizations due to RSV infection after the introduction of the pneumococcal vaccines^51^. In agreement with what reported by Miskill et al. this study found higher severity among bacterial-viral infection patients in terms of pneumonia, ICU admission, and case fatality rate than viral or bacterial infections^46,47^. Studies are needed to better understand the interactions between pathogenic bacteria and viruses during respiratory infections to facilitate the development of therapeutic or preventive strategies for improving patient outcomes.

Treatment of children with RSV is available using palivizumab for high-risk infants and children, however its short-term protection and high cost limit its effectiveness in reducing RSV prevalence^52,53^. There are several vaccine trials on mothers and infants in the pipeline, and it is expected that one or more of them will be approved within 1 to 3 years. These vaccines are using different techniques that can be used in the prevention of RSV in infants by active immunization^53^. A second strategy that is awaiting approval as the next step in preventing RSV is the extended half-life monoclonal antibodies. The medicine takes several months to be removed from the body, so a single dose will protect infants from RSV disease during the entire season, in spite of this, it is still expensive to make it widely available^49,50^. There is a need to develop preventive measures that are cost-effective and affordable for children living in developing countries where the largest burden of RSV exists^55^.

According to the American Academy of Pediatrics, vaccination against influenza is recommended for all children older than 6 months of age without medical contraindications for reducing the burden of respiratory illness. When children are at risk of influenza complications or have an inadequate immune response, antiviral chemoprophylaxis is recommended as an adjunct to vaccination^53^. However, these strategies may not be appropriate for developing countries. An intensive hand hygiene campaign conducted in Egypt in 2011 among primary school children proved successful in reducing laboratory-confirmed influenza^56^. Several National Hand Washing Campaigns were implemented in Egypt in collaboration between MoHP and WHO on World Hand Hygiene Day to raise community and children’s awareness of the importance of washing hands^57,58^.

### Limitations

Due to the fact that this study was conducted over a two-week period, the results are not representative of the different seasons among Egyptian children.

## Conclusions

Following two years of remission during the COVID-19 pandemic, a resurgence of common respiratory viruses, particularly RSV, was experienced among children in Egypt leading to a significant health problem. More hospitalizations with more severe disease courses and outcomes have been caused by the resurge of RSV and common respiratory viruses. The severity of viral infections has been exacerbated by bacterial superinfection. A comprehensive strategy includes enhancing hand hygiene among the community, especially children, enhancing ARI surveillance by broadening the laboratory testing panel, and encouraging physicians to use the surveillance results and the standard manual for ARI case management. Management of RSV infections should be directed to preventive measures, early detection, and proper case management in the absence of licensed vaccines and cost-effective treatment. Research is needed to understand the mechanisms underlying the decrease and rebound in seasonal non-SARS-CoV-2 viruses to inform decision-making to mitigate common respiratory epidemics and prepare for future pandemics. .

## Data Availability

The datasets generated and/or analyzed during the current study are not publicly available to protect study subjects’ privacy but are available from the corresponding author (OD) upon reasonable request.

## Abbreviations

RSV: Respiratory syncytial virus
SARI: Severe acute respiratory illness
RT-PCR: Real-time polymerase chain reaction
MoHP: Ministry of Health and Population
SARS-CoV-2: Severe acute respiratory coronavirus 2
ICU: Intensive care unit
NEDSS: National Infectious Diseases Surveillance
CPHL: Central Public Health Laboratories
LRTIs: Lower respiratory tract infections
